# 632. Impact of Substance Use and Withdrawal Management on Clinical Outcomes Following Burn Injury

**DOI:** 10.1093/jbcr/irag033.458

**Published:** 2026-04-06

**Authors:** Sebastian Kilcommons, Justin J Lee, Sharada Manchikanti, Joshua N Wong, Alexis Armour

**Affiliations:** University of Alberta, Edmonton, AB; University of Alberta, Edmonton, AB; University of Alberta, Edmonton, AB; University of Alberta, Edmonton, AB; University of Alberta, Edmonton, AB

## Abstract

**Introduction:**

Substance use and its impact on burn care are a growing concern, with notably a 50% increase in emergency calls for drug overdoses in recent years. Among burn patients, chronic substance use has been associated with increased hospital length of stay, complication rates, and post-injury mortality. Despite the high prevalence of addiction, there is a paucity of literature evaluating the effect of withdrawal management on outcomes among burn patients with illicit substance use. Our study outlined the implications of substance use for clinical outcomes following burn injury, and evaluated the impact of short-term, inpatient withdrawal treatment on modifying these outcomes.

**Methods:**

A chart review was conducted on all adult patients admitted to our center with burn injuries from 2022–2024. Primary outcome variables included substance use history, burn injury mechanism, and burn severity (TBSA and depth of injury). Secondary outcomes included length of stay, disposition from hospital, and complications, with inpatient withdrawal treatment (such as the CIWA, COWS, or regular opioid administration) as a variable. Analyses were conducted with an alpha of 0.05 using one-way ANOVA, Student’s t-tests, chi-squared tests, and/or nonparametric equivalents.

**Results:**

Among 576 patients included, 315 had a documented history of substance use. These patients were younger but with no significant gender difference between groups. Substance use was associated with a higher proportion of frostbite (29.2% vs 8.8%) and a lower proportion of thermal injuries (69.2% vs 83.9%). Substance use was not a factor in TBSA but was associated with a greater proportion of full-thickness burns. Patients with substance use had longer stays (23.8 vs 16.4 days), higher complication rates (17.2% vs 10.1%), and a 15-fold higher risk of unscheduled discharge compared to the control group. Of 250 patients using substances on admission, 171 (68.4%) received inpatient withdrawal management. Despite treatment of burn patients suffering from active withdrawal, these focused efforts did not result in any significant difference in clinical outcomes. Unscheduled discharge rates remained unchanged at 26% in both groups.

**Conclusions:**

Substance use in patients with burn injury is associated with higher rates of frostbite, deeper burns, longer hospital stays, higher complication rates, and a 15-fold increase in leaving against medical advice. Short term withdrawal treatment did not improve any of the clinical outcomes measured in our study. These findings illustrate the complexity of substance addiction among our patient population and support the need for long-term addiction treatment as part of the burn team.

**Applicability of Research to Practice:**

Our findings highlight disparities among burn patients with substance use to help inform future strategies to better support this vulnerable population.

**Funding for the study:**

N/A.

